# The role of gut endocrine cells in control of metabolism and appetite

**DOI:** 10.1113/expphysiol.2014.079764

**Published:** 2014-09-10

**Authors:** Helen E Parker, Fiona M Gribble, Frank Reimann

**Affiliations:** Cambridge Institute for Medical Research, Wellcome Trust/MRC Building, Addenbrooke's HospitalCambridge CB2 0XY, UK

## Abstract

•What is the topic of this review?

Gut hormones, especially glucagon-like peptide-1 (GLP-1), have beneficial effects in diabetes and obesity. Recent research addresses the underlying mechanisms of the secretion and action of GLP-1.

•What advances does it highlight?

The development of transgenic reporter mice with fluorescently tagged GLP-1-secreting cells has helped to characterize the molecular mechanisms underlying hormone secretion and has challenged the traditional classification of enteroendocrine cells by hormone expression alone. Recent adoption of this strategy to label GLP-1-receptor-positive cells has highlighted that peripheral and centrally released GLP-1 acts on a number of different targets, including a variety of neurons. Evidence for their role in glucose homeostasis and appetite control is discussed.

After food is ingested, nutrients pass through the gastrointestinal tract, stimulating the release of a range of peptide hormones. Among their many local, central and peripheral actions, these hormones act to mediate glucose metabolism and satiety. Indeed, it is the modification of gut hormone secretion that is considered partly responsible for the normalization of glycaemic control and the reduction in appetite seen in many patients after certain forms of bariatric surgery. This review describes recent developments in our understanding of the secretion and action of anorexigenic gut hormones, primarily concentrating on glucagon-like peptide-1 (GLP-1).

## Introduction

Over 20 different gut hormones are secreted from enteroendocrine cells scattered throughout the gut epithelial lining. Glucagon-like peptide-1 (GLP-1) is released from L-cells that are found along the length of the gut, with increasing density towards the colon. Glucagon-like peptide-1 plays an important role in promoting glucose homeostasis by augmenting insulin secretion, suppressing glucagon secretion and slowing gastric emptying, as well as by reducing food intake. Numerous studies have reported additional cardiovascular, renal and neurological1 effects of GLP-1 agonists, and the GLP-1 receptor (GLP1R) has been identified in the lung, kidney, blood vessels and sinoatrial cells of the heart (Richards *et al*. 2013; Pyke *et al*. 2014). However, the physiological importance of many of these findings is unclear.

**Figure 1:**
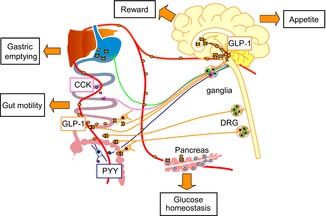
Glucagon-like peptide-1 (GLP-1), cholecystokinin (CCK) and peptide YY (PYY) are released from enteroendocrine cells in the intestinal lining and play an important role in glucose homeostasis and appetite control These hormones act locally, via afferent fibres close to their site of secretion, or after delivery around the body in the circulation. Recent studies investigating the site of GLP-1 receptor expression have given us further insights into the action of GLP-1. Glucagon-like peptide-1 acts on pancreatic *β*-cells and *δ*-cells to augment insulin and suppress glucagon secretion. Glucagon-like peptide-1 slows gastric emptying and gut motility, which is likely to involve both local and central signalling. Glucagon-like peptide-1 receptors are expressed in the gastric pylorus, myenteric ganglia and gut nerve fibres, as well as the ganglia of vagal and spinal afferents. Glucagon-like peptide-1 reduces food intake and appetite. Within the CNS, GLP-1 neurones are present in the nucleus of the solitary tract, which receives synaptic input from the vagus, and project to the appetite controlling regions of the hypothalamus, where GLP-1 receptors are also found. Glucagon-like peptide-1 receptors are also expressed in areas of the mesolimbic system associated with food motivation and reward. Abbreviation: DRG, dorsal root ganglion.

## Gut hormone secretion

Enteroendocrine cells have traditionally been categorized into distinct types based on their localization, morphology and hormonal signature. This view has been challenged recently by results generated from transgenic mouse lines expressing fluorescent reporters under the control of different gut peptide promoters. Such studies have enabled the purification of cell populations expressing GLP-1 (L-cells), glucose-dependent insulinotropic polypeptide (GIP; K-cells) or cholecystokinin (CCK; I-cells), suitable for transcriptomic analysis, and have demonstrated an unexpected degree of overlap between these cell types. The L- and K-cells in the small intestine, for example, were found additionally to express *CCK*, and comparison of their transcriptomes showed they were more similar to each other than to L-cells from the colon (Egerod *et al*. 2012; Habib *et al*. 2012). Although overlaps between enteroendocrine cell types had been noted in earlier immunohistochemical studies, the detection of cells expressing more than one hormone had previously been limited by the sensitivity of the antibodies used for cell identification (Mortensen *et al*. 2003; Theodorakis *et al*. 2006). The results suggest that cells expressing combinations of GLP-1, GIP, peptide YY (PYY), CCK, neurotensin and secretin derive from a common cell lineage and, with the high turnover of intestinal cells, raise the possibility that the endocrine system may be relatively adaptive.

The ability to isolate enteroendocrine cell populations has accelerated the identification of cell-specific receptors, ion channels and intracellular signalling pathways involved in the sensing of nutrients. L-Cells were found to be electrically excitable and to sense nutrient arrival downstream of electrogenic uptake or activation of G-protein-coupled receptors. The range of nutrients detected by enteroendocrine cells, and their associated sensors, include glucose (sodium-dependent glucose transporter 1, K_ATP_ channels and glucokinase), bile salts (GPBAR1), lipids (FFAR1, GPR120 and GPR119), short-chain fatty acids (FFAR2 and FFAR3) and amino acids (GPRC6A, CaSR, SNAT2 and B^0^AT1) and have been reviewed recently by our group (Ezcurra *et al*. 2013). Identifying the properties and functional roles of different enteroendocrine cell populations is crucial for the design of strategies to enhance gut peptide secretion from specific gut segments for the treatment of metabolic diseases.

## Glucagon-like peptide-1 and glucose metabolism

Glucagon-like peptide-1 and GIP are best known for mediating the incretin effect, whereby glucose administered orally triggers a much greater insulin response than a matched intravenous dose. Glucose-dependent insulinotropic polypeptide and GLP-1 receptor signalling in pancreatic *β*-cells activates adenylate cyclase, leading to the elevation of cAMP and augmentation of glucose-dependent insulin secretion. Given that GLP-1 retains effectiveness in people with type 2 diabetes and its action on insulin secretion is glucose dependent, thus limiting the incidence of hypoglycaemic side-effects, GLP-1 has proved an attractive therapeutic tool, and long-acting GLP-1 analogues have been front-line tools for the treatment of type 2 diabetes for the last decade.

Glucagon-like peptide-1 also inhibits glucagon secretion from *α*-cells, although whether this is a direct or an indirect effect is still controversial. In common with other attempts to identify the direct targets of GLP-1, uncertainties have arisen because of the inadequate specificity of antisera against GLP1R. An extensively validated GLP1R antibody has, however, demonstrated membrane-associated staining restricted to insulin-positive cells in human and monkey pancreas, with an absence from *α*-cells (Pyke *et al*. 2014). As an alternative to the use of antibodies for identifying GLP-1 targets, we generated transgenic reporter mice, in which cells expressing *glp1r* produce Cre recombinase and can be identified by fluorescent markers. Analysis of islets from these mice showed that *glp1r* is expressed in only ∼10% of *α*-cells, but is highly expressed in *β*-cells and somatostatin-producing *δ*-cells (Richards *et al*. 2013). The data thus support previous results from isolated, perfused rat pancreas, which showed that GLP-1-stimulated somatostatin secretion acts in paracrine manner to inhibit glucagon release from neighbouring *α*-cells (de Heer *et al*. 2008).

Glucagon-like peptide-1 is a strong regulator of gastrointestinal function, reducing gastric emptying, intestinal motility and gastric secretions (Edholm *et al*. 2010). These actions play an important part in the effect of GLP-1 on postprandial glycaemic excursions. The underlying mechanisms are not fully understood and likely to be complex. *Glp1r* was identified in scattered myenteric ganglia and fibres throughout the murine gut, and intraperitoneal administration of the GLP1R agonist exendin-4 induced Fos-like immunoreactivity in duodenal myenteric and submucosal neurons in fasted rats (Washington *et al*. 2010; Richards *et al*. 2013). *Glp1r* is also highly expressed in the gastric pylorus (Richards *et al*. 2013), and in healthy humans, exogenous GLP-1 increases pyloric tone, relaxes the fundus and reduces phasic contractions, resulting in slowed gastric emptying (Schirra *et al*. 2000, 2009). Central reflexes are likely to contribute to the control of gut motility, as suggested by the identification of *glp1r* in subpopulations of nodose and dorsal root ganglia cells, as well as in the central nervous system (CNS).

## Glucagon-like peptide-1 and appetite

Glucagon-like peptide-1 is part of a complex network of gut hormones and neurones involved in relaying information about food intake to the brain. The vagus nerve provides a pathway by which GLP-1, like several other gut hormones, can communicate with central appetite circuits. Vagal afferent fibres project peripherally to the visceral organs, including much of the gastrointestinal system, and centrally to the brainstem, from which signals are relayed to the hypothalamus. The finding that the effects of peripherally administered GLP-1 on food intake were ablated after bilateral subdiaphragmatic total vagotomy in rats demonstrated the significance of vagal signalling in appetite control (Abbott *et al*. 2005). Indeed, GLP-1 directly stimulates activity in the afferent vagus and augments Ca^2+^ responses in subpopulations of nodose ganglion cell bodies *in vitro* (Bucinskaite *et al*. 2009; Richards *et al*. 2013).

In practice, the responsiveness of the vagus may vary according to the nutritional status, as shown by an altered balance in the expression of orexigenic *versus* anorexigenic signalling machinery (Dockray & Burdyga, 2011), predominantly influenced by CCK but also by leptin and apolipoprotein AIV (Dockray, 2013). In rodent models, high-fat diet-induced obesity also reduces vagal afferent sensitivity by disrupting the expression of receptors such as *glp1r* (Daly *et al*. 2011; Duca *et al*. 2013). Interestingly, we identified cells expressing *glp1r* in dorsal root ganglia, suggesting that spinal sensory neurones might represent an alternative route of communication to the CNS. This raises the possibility that GLP-1 released from proximal compared with distal L-cells might trigger different central responses, depending on the neural circuitry recruited. The idea that endogenous GLP-1 released from different sites might not be functionally equivalent is an important factor to consider in the design of new therapeutics, and may be relevant to the interpretation of bariatric surgery outcomes which favour GLP-1 release from more distal L-cell populations.

As GLP-1 and GLP1R are also produced in various regions of the CNS, the role of gut-derived (as opposed to centrally derived) GLP-1 has been difficult to define. Within the CNS, GLP-1 neurones are found predominantly in the nucleus of the solitary tract, which receives synaptic input from vagal afferent fibres. These neurones are responsive to leptin and CCK but not to PYY or GLP-1 itself (Hisadome *et al*. 2010, 2011). Glucagon-like peptide-1 neurones in the nucleus of the solitary tract project to the hypothalamus, where *glp1r* is expressed in the arcuate and paraventricular nuclei, raising the possibility that gut-derived GLP-1, signalling via the vagus, may result in the release of central GLP-1 (Llewellyn-Smith *et al*. 2011; Richards *et al*. 2013).

A study using peripherally or centrally injected exendin-(9–39), a GLP1R antagonist, prior to peripherally or centrally injected GLP-1 demonstrated that the reduction in food intake due to peripheral GLP-1 was blocked only by peripheral exendin-(9–39), whereas the effect of central GLP-1 was blocked only by central exendin-(9–39). This indicated that in its effects on appetite, gut-derived GLP-1 acts predominantly via GLP-1 receptors accessible from the periphery (Williams *et al*. 2009). *Glp1r* expression has also, however, been found in areas of the CNS that are accessible to circulating hormones, notably the arcuate nucleus and area postrema. Indeed, Fos activation in the area postrema was more strongly induced by peripheral than central exendin-4 administration (Yamamoto *et al*. 2003). Endogenous gut-derived GLP-1, with its short circulating half-life, is unlikely to penetrate the blood–brain barrier, but more stable GLP-1 analogues appear able to penetrate deeper regions of the CNS (Kastin *et al*. 2002; Hunter & Hölscher, 2012)

In addition to the anorexigenic effects mediated via brainstem–hypothalamic circuits, GLP-1 signalling may also play a role in food reward and motivation. *Glp1r* is expressed in the ventral tegmental area and nucleus accumbens, areas of the mesolimbic system associated with feelings of reward and desire. In rats, GLP-1 or exendin-4 injected peripherally reduced palatable food intake and reward-motivated behaviour, and this effect was still seen when exendin-4 was microinjected directly into the ventral tegmental area (Dickson *et al*. 2012; Mietlicki-Baase *et al*. 2013). In addition, the antagonist exendin-(9–39) injected into the nucleus accumbens increased meal size and palatability of sucrose solutions (Dossat *et al*. 2013). Interestingly, direct stimulation of ventral tegmental area GLP-1 receptors in rats also reduced alcohol intake (Shirazi *et al*. 2013).

## Conclusions

Glucagon-like peptide-1-based therapies are effective treatments for type 2 diabetes, and mimetics of GLP-1 are under evaluation as anti-obesity agents. As well as having glucose-lowering effects mediated by the pancreas, benefits include weight loss and slowed gastric emptying. Many of the reported actions of GLP-1 are not fully understood, but recent confirmation of the expression of *glp1r* in pancreatic islet cells and the peripheral and central nervous system will aid the clarification of GLP-1 physiology and pharmacology.

The enhancement of endogenous GLP-1 release to treat diabetes is an alternative therapeutic strategy currently under investigation. As enteroendocrine cells are now recognized to express a spectrum of hormonal mediators, targeting the gut endocrine system could release a soup of metabolically active hormones with anorexigenic as well as incretin effects and mimic some of the physiological responses to gastric bypass surgery. Analyses of isolated enteroendocrine cell populations have identified sensory pathways that might be suitable for pharmacological targeting. Further insights into the GLP-1-secreting enteroendocrine cell population and GLP1R expression and signalling will allow us to exploit the properties of this gut hormone to the fullest.

## Call for comments

Readers are invited to give their opinion on this article. To submit a comment, go to: http://ep.physoc.org/letters/submit/expphysiol;99/9/1154
